# Effectiveness of adjuvant traditional Chinese medicine on macrovascular invasion in patients with hepatocellular carcinoma: a real-world propensity score-matched study

**DOI:** 10.3389/fphar.2024.1353720

**Published:** 2024-02-23

**Authors:** Huiwen Yan, Xinhui Wang, Lihua Yu, Xiaoli Liu, Fengna Yan, Yuqing Xie, Qing Pu, Zhiyun Yang

**Affiliations:** Center of Integrative Medicine, Beijing Ditan Hospital Affiliated to Capital Medical University, Beijing, China

**Keywords:** anticancer Chinese pattern medicine, complementary alternative medicine, hepatocellular carcinoma, macrovascular invasion, traditional Chinese medicine

## Abstract

The study aimed to investigate the potential of traditional Chinese medicine (TCM) in reducing the risk of macrovascular invasion (MVI) in Chinese patients with hepatocellular carcinoma (HCC). This retrospective analysis involved 2,267 HCC patients treated at our hospital. Propensity score (PS) matching was used to compare TCM users (n = 485) with non-users (n = 485) in terms of age, Barcelona Clinic Liver Cancer (BCLC) staging, type of treatment, and AFP. The impact of TCM on the hazard ratio (HR) of MVI was evaluated using a Cox multivariate regression model. The efficacy of TCM therapy on MVI was further examined using the log-rank test. The analysis revealed that TCM medication was a significant protective factor for MVI in HCC patients, as evidenced by the Cox analysis (adjusted HR = 0.496, 95% CI: 0.387–0.635, *p* < 0.001). After PS matching, the Kaplan-Meier curve demonstrated a lower occurrence rate of MVI in TCM users compared to non-users. The study findings suggest that TCM treatment has the potential to decrease the incidence of MVI in HCC patients, irrespective of etiology, BCLC staging, liver function, or treatment type. Notably, as the use of TCM increased, the percentage of MVI in patients showed a gradual decrease, indicating the potential of TCM therapy as a successful strategy for preventing MVI.

## Introduction

According to recent international research statistics, the global incidence of hepatocellular carcinoma (HCC) in 2020 was reported to be 905,600, resulting in 830,100 deaths—an increase of 48,500 cases compared to 2018. Notably, China accounted for over half of the new cases and fatalities (Sung et al., 2021). Numerous patients were in middle to late stages of HCC when diagnosed because of the liver’s robust ability to compensate, the absence of early HCC symptoms, and the disease’s quick progression (Zhong et al., 2017). Microvascular invasion and macrovascular invasion (MVI) are two types of vascular invasion. The growth of microvascular invasion over time leads to MVI, which is a sign of advanced HCC, according to BCLC recommendations. Research has shown that patients with HCC had an incidence of MVI up to 62.2%. Median survival times of patients with MVI are only up to 4 months once a tumor thrombus has formed in the portal vein (Forner et al., 2018). As a result, MVI is frequently present in HCC patients and the prognosis once it occurs is very poor.

The field of HCC treatment is characterized by multi-disciplinary participation and the coexistence of multiple treatment methods. The common treatment methods include hepatectomy, liver transplantation, ablation, transarterial chemoembolization (TACE), radiotherapy, systematic anti-tumor therapy and so on. Choosing reasonable treatment methods for patients with different stages of HCC can maximize the curative effect (Omata et al., 2017). TACE, radiofrequency ablation (RFA), and surgical various surgical procedures (transplantation of the liver, resection) and other treatments are available for HCC patients without MVI. RFA and TACE are common treatment approaches that can be used for various tumor stages among them (Llovet et al., 2021a). Vascular endothelial growth factor (VEGF) is a cytokine. After treatment with RFA (Guan et al., 2015; Markezana et al., 2021) and TACE (Petrillo et al., 2018), the concentration of VEGF in HCC patients increases. The growth of tumors and vascular invasion can be accelerated by VEGF by impairing T cell function, increasing the recruitment of T regulatory cells, myelogenous suppressor cells (MDSC), and mast cells, and preventing dendritic cell development and activation (Fukumura et al., 2018; Yang et al., 2018). Interestingly, as an effective multi kinase inhibitor against the VEGF receptor, sorafenib combined with TACE is considered to have synergistic effect in the treatment of HCC. Unfortunately, throughout the past 10 years, a number of multicenter randomised controlled trials (Lencioni et al., 2016; Meyer et al., 2017; Park et al., 2019) failed to show the BCLC and TACE combination’s anticipated synergistic effects as a unique treatment for patients with advanced disease. Brivanib, a humanized monoclonal antibody against VGEF, has become a widely administered choice for anti-angiogenesis therapy (Galanopoulos et al., 2016). Unfortunately, TACE combined with brivanib and tyrosine kinase inhibitors (such as orantinib) also showed negative treatment benefits (Kudo et al., 2014; Kudo et al., 2018). Therefore, the ideal candidate for TACE combined with molecular targeted drugs for the treatment of HCC or biomarkers for predicting MVI remain to be unequivocally identified. In order to reduce the incidence of MVI and weaken the adverse reactions caused by treatment, patients are given supplementary replacement therapy (Shi et al., 2017).

In China, traditional Chinese medicine (TCM) is the predominant alternative therapy for individuals with chronic liver disease. Studies have demonstrated that TCM can prevent tumour escape, progression, and metastasis as well as liver cancer growth in the particular immunological tolerance setting of HCC (Wang et al., 2015; Jia and Wang, 2020). It has been found that TCM can block the mTOR/HIF-1α/VEGF signaling pathway which effectively inhibits the migration, invasion and angiogenesis of colon cancer cells (Peng et al., 2018). So far, TCM derived metabolites have shown great potential in slowing tumor progression by downregulating VEGF-related signal pathways (Zhang C. et al., 2018). There has been, however, only limited clinical research on MVI and anti-tumor Chinese patent medication in patients with HCC. Assessing the influence of TCM adjuvant therapy in combination with conventional chemoradiotherapy on the occurrence of MVI, the research analyzed the impact of TCM treatment on HCC from various causes. The findings of this study offer robust guidelines for the utilization of adjuvant TCM therapy for patients with HCC.

## Methods

### Patients and follow-up

**FIGURE 1:**
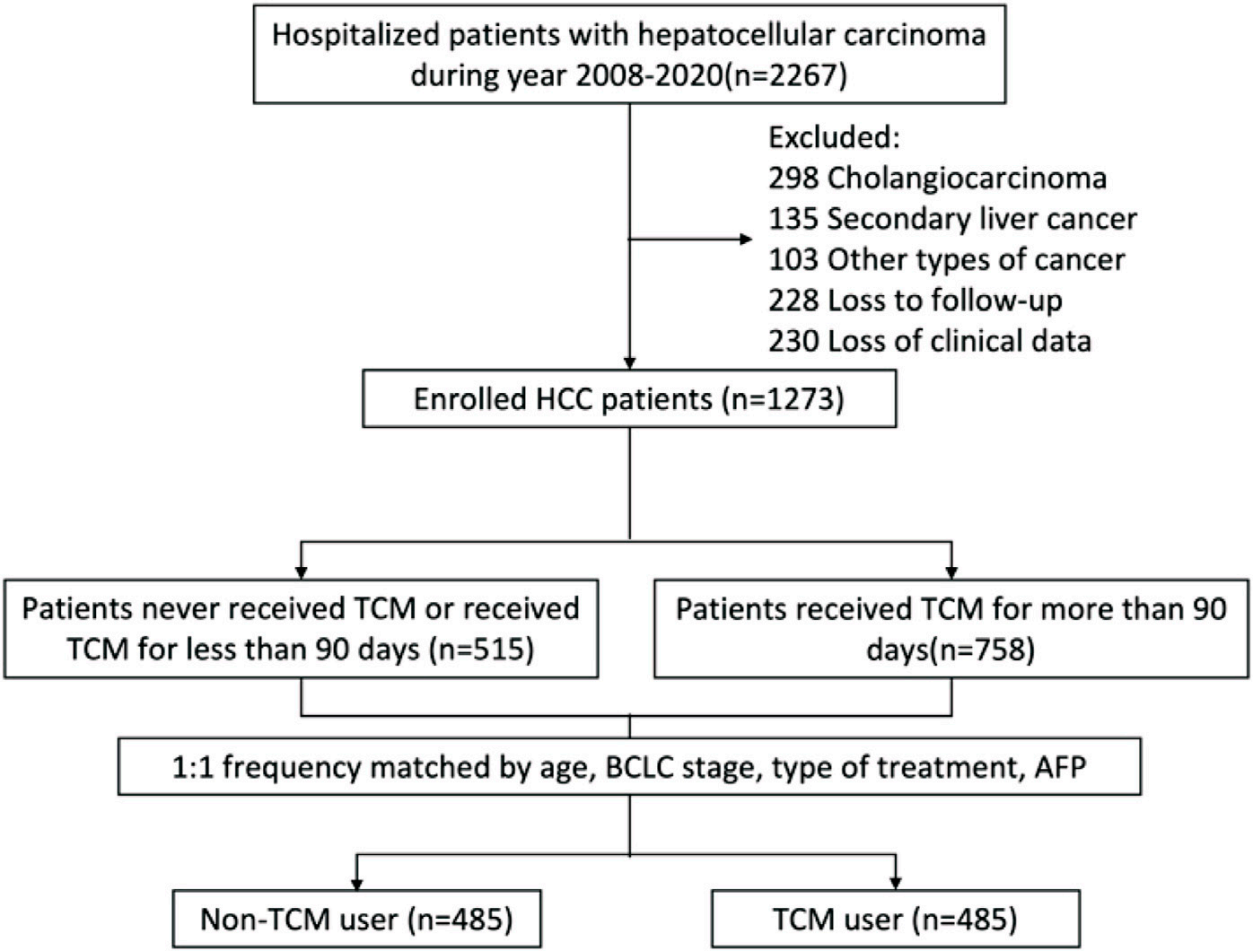
An overview of the identification and recruitment procedures for the hepatocellular carcinoma study cohort. TCM, traditional Chinese medicine.

In our hospital, 2,267 individuals were diagnosed with primary liver cancer between January 2009 and December 2020. The research project received approval from the ethical committee at Capital Medical University’s Beijing Ditan Hospital, along with a waiver of informed consent. All procedures were conducted in accordance with the 2008 Helsinki Declaration and the ethical standards outlined by the relevant national and institutional committees governing human research. Since the observation population was patients without MVI, we also selected patients for inclusion in minimally invasive treatment recommended by the guidelines (Omata et al., 2017). The study’s inclusion criteria comprised: 1) primary liver cancer; 2) absence of vascular invasion; 3) ages ranging from 18 to 75; and 4) history of receiving TACE, RFA, or a combination of both. Exclusion criteria included cholangiocarcinoma (n = 298 patients), subsequent liver cancer (n = 135), co-occurrence with other tumor types (n = 103), lack of follow-up (n = 228 patients), and inadequate clinical records (n = 230 patients). As depicted in Figure 1, a total of 1,273 patients was included in the study and categorized into two groups: those utilizing TCM extensively and those who did not. The groups were matched in a 1:1 ratio based on age, BCLC stage, type of treatment, and AFP levels. The primary outcome of interest was the occurrence of MVI in patients with HCC. The observation period for each patient extended from the commencement of their participation in the study to the development of MVI or the date of 31 December 2021, whichever came first.

### Diagnostic criteria for HCC and MVI

HCC is diagnosed by histopathology at first, and then by clinical diagnosis. Two imaging methods (liver ultrasound, CT, hepatic angiography and MRI) indicate the lesions, or one imaging method indicates the lesions with alpha-fetoprotein (AFP) ≥ 400 ng/mL (Llovet et al., 2021b). Using enhanced CT or enhanced MRI imaging, MVI was diagnosed when filling defects occurred in the portal or hepatic veins, inferior vena cava and when embolus enhancement was similar to that of HCC (Shah et al., 2007).

### The treatment of TCM and the composition of each TCM formulation

After diagnosing HCC, it is necessary to have a medical record indicating that the patient has undergone a minimum of 3 months of TCM treatment. The TCM treatment primarily consists of Chinese patent medicines sanctioned by the China State Food and Drug Administration (SFDA) and commonly used for treating HCC. These include Fufang Banmao (Z52020238), Jinlong (Z10980041), Kanglixin (Z20025075), Ganfule (Z20060389) capsules, and Huaier Granule (Z20000109)(Li K. et al., 2022). We follow the ConPhyMP statement to ensure the scientific accuracy and credibility of the article (Heinrich et al., 2022). All species in these proprietary Chinese medicines are named according to the rules established by Rivera et al. (Rivera et al., 2014). Their main chemical composition and analytical methods are provided in Table 1. Fufang Banmao Capsule comprises 11 traditional Chinese medicines, with Cantharidin being the primary material present at a minimum concentration of 0.0175 mg/g (Naz et al., 2020). Jinlong Capsule is an animal-derived Chinese patent medicine prepared using modern biochemical separation technology involving low temperature and freezing. It consists of 17 amino acids and a variety of bioactive peptides, with the active principles accounting for 31.2% of the capsule content (Li C. et al., 2022). Kanglixin Capsule, as reported in previous studies, contains 9 traditional Chinese medicines ranging in concentration from 0.562 mg/g to 3.874 mg/g (Li and Kong, 2017). Ganfule Capsule is a compound preparation consisting of 21 traditional Chinese medicines. The active metabolites in Ganfule Capsule account for 25% of its content (Xu et al., 2022). Huaier granules primarily contain *Trametes robiniophila* Murr, a medicinal fungus rich in various organic metabolites and more than 10 minerals. The active metabolite in Huaier Granule is polysaccharide protein, with a protein content reported to be no less than 71.5 mg/g (Pan et al., 2019).

**TABLE 1:** Common TCM for the treatment of HCC.

Name	Components	Analytical methods	Main chemical substances
Fufang Banmao Capsule (Z52020238)	Mylabris (Banmao), *Panax ginseng* C.A.Mey. [Araliaceae; Ginseng radix et rhizome], *Astragalus mongholicus* Bunge [Fabaceae; Astragalus mongholicus radix], *Eleutherococcus senticosus* (Rupr. & Maxim.) [Araliaceae; Acanthopanacis senticosi radix et rhizoma seu caulis], *Sparganium stoloniferum* (Buch.-Ham. ex Graebn.) Buch.-Ham. ex Juz. [Typhaceae; Sparganii rhizome], *Scutellaria barbata* D.Don [Lamiaceae; Scutellariae barbatae herba], *Curcuma aromatica* Salisb. [Zingiberaceae; Curcumae rhizome], *Cornus officinalis* Siebold & Zucc. [Cornaceae; Corni fructus], *Ligustrum lucidum* W.T.Aiton [Oleaceae; Ligustri lucidi fructus], Bear bile powder, *Glycyrrhiza glabra* L. [Fabaceae; Glycyrrhizae radix et rhizome]	GC	Cantharidin, ginsenosides Rg1, ginsenosides Rg3, ginsenosides Rb1, ginsenosides Re, astragaloside A, isofraxidin
Jinlong Capsule (Z10980041)	Fresh gecko, Fresh multibanded krait, Fresh long-nosed pit viper	UPLC	Histidine, serine, arginine, glycine, aspartic acid, glutamate, threonine, alanine, proline, cystine, lysine, tyrosine, methionine, valine, isoleucine, leucine, phenylalanine
Kanglixin Capsule (Z20025075)	*Ferula sinkiangensis* K.M.Shen [Apiaceae; Ferulae resina], Aspongopus (Jiuxiangchong), *Rheum officinale* Baill. [Polygonaceae; Rheum officinale Baill. radix et rhizoma Rhei], *Curcuma longa* L. [Zingiberaceae; Curcumae longae rhizome], *Terminalia chebula* Retz. [Combretaceae; Terminalia chebula Retz. var. tomentella (Kurz) C. B. Clarke], *Syzygium aromaticum* (L.) Merr. & L.M.Perry [Myrtaceae; Syzygium aromaticum flos], *Dolomiaea costus* (Falc.) Kasana & A.K.Pandey [Asteraceae; Dolomiaea costus radix], Cordyceps Sinensis (Dongchongxiacao)	HPLC	Costunolide, dehydrocostus lactone, aloe-emodin, rhein, emodin, physcion, bisdemethoxycurcumin, demethox-ycurcumin, curcumin
Ganfule Capsule (Z20060389)	*Codonopsis pilosula* (Franch.) Nannf. [Campanulaceae; Codonopsis pilosula radix], Trionycis Carapax, *Paris yunnanensis* Franch. [Melanthiaceae; Paridis rhizome], *Atractylodes macrocephala* Koidz. [Asteraceae; Atractylodis macrocephalae rhizome], *Astragalus mongholicus* Bunge [Fabaceae; Astragali mongholici radix], *Citrus reticulata* Blanco [Rutaceae; Citri reticulatae Pericarpium], Eupolyphaga steleophaga, *Rheum officinale* Baill. [Polygonaceae; Rheum officinale Baill. radix et rhizoma Rhei], *Juglans regia* L. [Juglandaceae; Persicae semen], *Scutellaria barbata* D.Don [Lamiaceae; Scutellariae barbatae herba], *Patrinia scabiosifolia* Link [Caprifoliaceae; Patrinia scabiosifolia herba], *Smilax glabra* Roxb. [Smilacaceae; Smilacis glabrae rhizoma], *Coix lacryma-jobi* L. [Poaceae; Coix lacryma-jobi semen], *Curcuma longa* L. [Zingiberaceae; Curcumae radix], *Biancaea sappan* (L.) Tod. [Fabaceae; Sappan Lignum], Ostreae Concha, *Artemisia scoparia* Waldst. & Kit. [Asteraceae; Artemisiae scopariae herba], *Akebia trifoliata* (Thunb.) Koidz. [Lardizabalaceae; Akebiae caulis], *Cyperus rotundus* L. [Cyperaceae; Cyperi rhizome], *Aquilaria sinensis* (Lour.) Spreng. [Thymelaeaceae; Aquilariae lignum resinatum], *Bupleurum chinense* DC. [Apiaceae; Bupleuri radix]	UPLC-Q-TOF-MS	Chlorogenic acid, amygdalin, 3′-deoxysappanone A, 10-O-Methylprotosappanin B, scutellarin, narirutin, hesperidin, hesperetin, nobiletin, 3,3′,4′,5,6,7,8-heptamethoxyflavone, saikosaponin A, Saikogenin C and astragaloside I
Huaier Granule (Z20000109)	*Trametes robiniophila* Murr	UHPLC-MS	Proteoglycan,1β-Hydroxyalantolactone, 2-Aminoisobutyric acid, 2-Hydroxybutanoic acid, 2-Isopropyl-3-oxosuccinate, 2′-O-Methyladenosine, 2-Picolinic acid, 3,4-Dihydroxyphenylacetaldehyde, 3-Ethyl-1,2-benzenediol

GC, gas chromatography; HPLC, liquid chromatography–mass spectrometry; UPLC, ultra-performance liquid chromatography; UPLC-Q-TOF-MS, ultra-performance liquid chromatography to quadrupole time-of-flight mass spectrometry; UHPLC-MS, ultrahigh-pressure liquid chromatography coupled with tandem mass spectrometry.

### Demographic and clinical data

Data retrieved from medical records were: patient ages; gender; family HCC history; smoking or alcohol use; hypertension; diabetes; hyperlipidemia; coronary disease; cirrhosis, etiology; model for end-stage liver disease (MELD) scores; Child-Pugh stage; types of treatment; and hepatitis B virus related indicators such as HBeAg and HBV-DNA. Tumor-related indicators included: BCLC staging; multiple tumors and their sizes. White blood cell counts (WBC), hemoglobin concentration (HGB), platelet counts (PLT), aspartate aminotransferase (AST), total bilirubin (TBIL), albumin (ALB), γ-glutamyl transferase (γ-GGT), prothrombin activity (PTA), alanine aminotransferase (ALT), international normalised ratio (INR), AFP and C-reactive protein (CRP) tests were carried out in the hospital laboratory.

### Statistical analysis

Statistical analyses were carried out using R ver. 4.0.1. To check for normalcy, the Kolmogorov-Smirnov method was employed. A *t*-test was employed to evaluate quantitative data that was normally distributed with the results presented as means ± SDs. The Mann-Whitney *U* test was employed to make comparisons between non-normal distributions using median (m) and quartile range (QR) (m, QR). Qualitative data differences were evaluated using a χ^2^ test, and the results are expressed as frequencies/numbers. In order to examine the independent risk variables for vascular invasion in HCC patients, univariate and multivariate Cox analyses were utilised. Kaplan-Meier was utilized to produce the patient occurrence curve, and a log-rank test to compare the occurrence rates of MVI in TCM users and non-TCM users. Forest plots were built to compare the 2 groups for the 1-year hazard ratio (HR) of vascular invasion for each aetiology, BCLC stage, Child-Pugh staging, method of treatment, and varied AFP levels. Sankey plots were created using networkD3 software to demonstrate the MVI outcomes for patients who used TCM at various intervals. A *p*-value below 0.05 indicates statistical significance.

### Propensity score matching

We employed propensity score matching to adjust variables and utilized a logistic regression model to calculate the propensity score, which indicates the likelihood of each patient receiving TCM treatment. The matching propensity score model incorporated key variables including age, BCLC stage, type of treatment, and AFP. Utilizing the nearest neighbor method with a 1:1 ratio, a caliper width of 0.05 was applied without replacement. The standardized mean difference (SMD) was used to assess the balance of variables between groups before and after matching, with values below 0.10 indicating balance.

## Results

### Baseline characteristics of study participants

Out of a total of 1,273 HCC patients, 758 (59.54%) received TCM treatment for >90 days after their diagnosis and 515 (40.46%) did not. Prior to PS matching, patients with HBV-DNA levels under 500 IU/mL were more likely to take TCM. TCM users were more prevalent in BCLC stage 0-A than non-users were, although fewer patients were in stage B and there were more single tumors with diameters <5 cm (*p* < 0.001). Additionally, PLT, AST and γ-GGT levels were lower. The proportion of patients an AFP concentration less than 400 ng/mL was greater (*p* < 0.05), as was the albumin level. The number of TCM users who had received TACE was lower, and they were given combination TACE + RFA treatment (*p* < 0.001). The clinical measures of WBC, ALT, PTA, and INR were all within the normal range, despite statistical variations being found between TCM and non-TCM users. (Table 2). PS matching was carried out as a result of the patients’ baseline heterogeneity between those who used TCM and those who did not. Following the analysis, tumor features and available treatments were virtually identical for TCM and non-TCM users (Supplementary Table S1).

**TABLE 2:** Demographics and clinical features of patients with hepatocellular carcinoma.

		Total *n* = 1,273 (%)	Non-TCM users *n* = 515 (%)	TCM users *n* = 758 (%)	*p-*value
Patient features					
Age, years (mean ± SD)		57.09 ± 10.03	57.06 ± 9.90	57.11 ± 1 0.13	0.931
Gender	Female	292 (22.9)	116 (22.5)	176 (23.2)	0.825
	Male	981 (77.1)	399 (77.5)	582 (76.8)	
Family history of HCC	No	1,224 (96.2)	492 (95.5)	732 (96.6)	0.427
	Yes	49 (3.8)	23 (4.5)	26 (3.4)	
History of smoking	No	766 (60.2)	314 (61.0)	452 (59.6)	0.674
	Yes	507 (39.8)	201 (39.0)	306 (40.4)	
History of alcohol use	No	803 (63.1)	324 (62.9)	479 (63.2)	0.966
	Yes	470 (36.9)	191 (37.1)	279 (36.8)	
Hypertension	No	922 (72.4)	386 (75.0)	536 (70.7)	0.11
	Yes	351 (27.6)	129 (25.0)	222 (29.3)	
Diabetes	No	986 (77.5)	405 (78.6)	581 (76.6)	0.443
	Yes	287 (22.5)	110 (21.4)	177 (23.4)	
Hyperlipidemia	No	1,170 (91.9)	469 (91.1)	701 (92.5)	0.422
	Yes	103 (8.1)	46 (8.9)	57 (7.5)	
Coronary	No	1,240 (97.4)	499 (96.9)	741 (97.8)	0.44
	Yes	33 (2.6)	16 (3.1)	17 (2.2)	
Cirrhosis	No	135 (10.6)	63 (12.2)	72 (9.5)	0.144
	Yes	1,138 (89.4)	452 (87.8)	686 (90.5)	
Etiology	HBV	1,014 (79.7)	396 (76.9)	618 (81.5)	0.1
	HCV	120 (9.4)	58 (11.3)	62 (8.2)	
	Alcohol abuse	101 (7.9)	41 (8.0)	60 (7.9)	
	Other	38 (3.0)	20 (3.9)	18 (2.4)	
HBeAg	Negative	673 (52.9)	266 (51.7)	407 (53.7)	0.119
	Positive	341 (26.8)	130 (25.2)	211 (27.8)	
	NA	259 (20.3)	119 (23.1)	140 (18.5)	
HBV-DNA, IU/mL	<5 00	556 (43.7)	191 (37.1)	365 (48.2)	<0.001
	≥500	458 (36.0)	205 (39.8)	253 (33.4)	
	NA	259 (20.3)	119 (23.1)	140 (18.5)	
MELD scores		4.31 (1.63, 6.66)	4.40 (1.67, 6.80)	4.22 (1.62, 6.59)	0.289
Child-Pugh stage	A	856 (67.2)	338 (65.6)	518 (68.3)	0.343
	B	417 (32.8)	177 (34.4)	240 (31.7)	
BCLC staging	0-A	671 (52.7)	220 (42.7)	451 (59.5)	<0.001
	B	602 (47.3)	295 (57.3)	307 (40.5)	
Tumor multiplicity	Solitary	786 (61.7)	276 (53.6)	510 (67.3)	<0.001
	Multiple	487 (38.3)	239 (46.4)	248 (32.7)	
Tumor size, cm	<5	994 (78.1)	372 (72.2)	622 (82.1)	<0.001
	≥5	279 (21.9)	143 (27.8)	136 (17.9)	
Laboratory data					
WBC (10^9^/L)		4.35 (3.11, 5.85)	4.59 (3.12, 6.08)	4.17 (3.11, 5.69)	0.021
HGB (g/L)		132.40 (116.80, 145.00)	131.00 (115.00, 143.10)	134.00 (117.45, 145.60)	0.082
PLT (10^9^/L)		96.30 (62.30, 146.00)	105.20 (64.70, 155.80)	92.00 (61.80, 140.35)	0.002
ALT (U/L)		30.50 (21.60, 49.10)	32.40 (22.30, 53.20)	30.30 (21.20, 47.00)	0.042
AST (U/L)		35.40 (25.90, 54.30)	38.00 (27.00, 61.05)	34.20 (25.33, 50.00)	<0.001
TBIL (µmol/L)		16.60 (11.70, 23.10)	16.60 (11.40, 23.40)	16.55 (12.10, 22.67)	0.824
ALB (g/L)		37.60 (33.50, 41.00)	37.20 (32.70, 40.75)	38.00 (34.00, 41.20)	0.013
γ-GGT (U/L)		45.00 (28.80, 91.20)	58.40 (33.35, 111.60)	44.90 (26.95, 73.60)	<0.001
PTA (%)		80.70 (70.10, 91.00)	79.00 (69.30, 90.00)	81.70 (71.00, 92.00)	0.025
INR		1.09 (1.02, 1.19)	1.10 (1.02, 1.21)	1.09 (1.01, 1.18)	0.018
AFP (ng/mL)	<400	1,028 (80.8)	383 (74.4)	645 (85.1)	<0.001
	≥400	245 (19.2)	132 (25.6)	113 (14.9)	
CRP (mg/L)		3.20 (3.20, 6.62)	3.20 (3.20, 17.70)	3.20 (3.00, 3.20)	<0.001
Type of treatment					
TACE	No	858 (67.4)	292 (56.7)	566 (74.7)	<0.001
	Yes	415 (32.6)	223 (43.3)	192 (25.3)	
RFA	No	1172 (92.1)	474 (92.0)	698 (92.1)	1
	Yes	101 (7.9)	41 (8.0)	60 (7.9)	
TACE + RFA	No	516 (40.5)	264 (51.3)	252 (33.2)	<0.001
	Yes	757 (59.5)	251 (48.7)	506 (66.8)	

### Univariate and multivariate statistical analyses

The impact of baseline indicators on the incidence of 1-year MVI was evaluated by employing a multivariate Cox proportional hazards model. Independent protective factor for the 1-year incidence of MVI in HCC patients were taking TCM (adjusted HR = 0.4941, 95% CI: 0.3853–0.6336, *p* < 0.001) and high PTA (adjusted HR = 0.9893, 95% CI: 0.9807–0.9980, *p* = 0.016). Compared to the combination therapy of TACE and RFA, both only TACE and only RFA were independent risk factors, TACE (adjusted HR = 1.5771, 95% CI: 1.2238–2.0324, *p* < 0.001), RFA (adjusted HR = 2.1287, 95% CI: 1.4239–3.1824, *p* < 0.001). In addition, Cirrhosis (adjusted HR = 1.8541, 95% CI: 1.1379–3.0213, *p* = 0.013), size of tumor ≥5 cm (adjusted HR = 1.9713, 95% CI: 1.5209–2.5550, *p* < 0.001), AFP ≥400 ng mL (adjusted HR = 1.8929, 95% CI: 1.4668–2.4428, *p* < 0.001), and a high serum CRP concentration (adjusted HR = 1.0053, 95% CI: 1.0024–1.0083, *p* < 0.001) were independent risk factors (Table 3).

**TABLE 3:** Factors associated with the development of MVI of patients with hepatocellular carcinoma.

Variables	Univariate analysis		Multivariate analysis	
	HR (95% CI)	*p*-value	HR (95% CI)	*p*-value
TCM	0.3788 (0.2991–0.4798)	<0.001	0.4941 (0.3853, 0.6336)	<0.001
Age >50 years	0.7964 (0.6076–1.0439)	0.099		
Gender (male)	1.2845 (0.9575–1.7232)	0.095		
Family history of HCC	0.8548 (0.4549–1.6063)	0.626		
History of smoking	1.2558 (0.9955–1.5841)	0.055		
History of alcohol use	1.2467 (0.9863–1.5758)	0.065		
Cirrhosis	1.8591 (1.1537–2.9958)	0.011	1.8541 (1.1379, 3.0213)	0.013
Tumor multiplicity	1.5005 (1.1906–1.8911)	<0.001		
Tumor size ≥5 cm	2.7205 (2.1468–3.4473)	<0.001	1.9713 (1.5209, 2.5550)	<0.001
HBV-DNA, positive	1.5666 (1.2108–2.0270)	<0.001		
Type of treatment				
TACE	2.2170 (1.7360–2.8310)	<0.001	1.5771 (1.2238, 2.0324)	<0.001
RFA	1.8825 (1.2630–2.8060)	0.002	2.1287 (1.4239, 3.1824)	<0.001
TACE + RFA	Reference			
WBC (10^9^/L)	1.0430 (0.9945–1.0938)	0.083		
HGB (g/L)	0.9919 (0.9872–0.9968)	0.001		
PLT (10^9^/L)	1.0011 (0.9994–1.0028)	0.214		
ALT (U/L)	1.0014 (0.9997–1.0031)	0.103		
AST (U/L)	1.0022 (1.0006–1.0039)	0.006		
TBIL (µmol/L)	1.0013 (0.9980–1.0045)	0.456		
ALB (g/L)	0.9698 (0.9508–0.9891)	0.002		
PTA (%)	0.9848 (0.9774–0.9923)	<0.001	0.9893 (0.9807, 0.9980)	0.016
CRP (mg/L)	1.0090 (1.0065–1.0116)	<0.001	1.0053 (1.0024, 1.0083)	<0.001
AFP ≥400 ng/mL	2.3999 (1.8755–3.0710)	<0.001	1.8929 (1.4668, 2.4428)	<0.001

### Survival analysis

**FIGURE 2:**
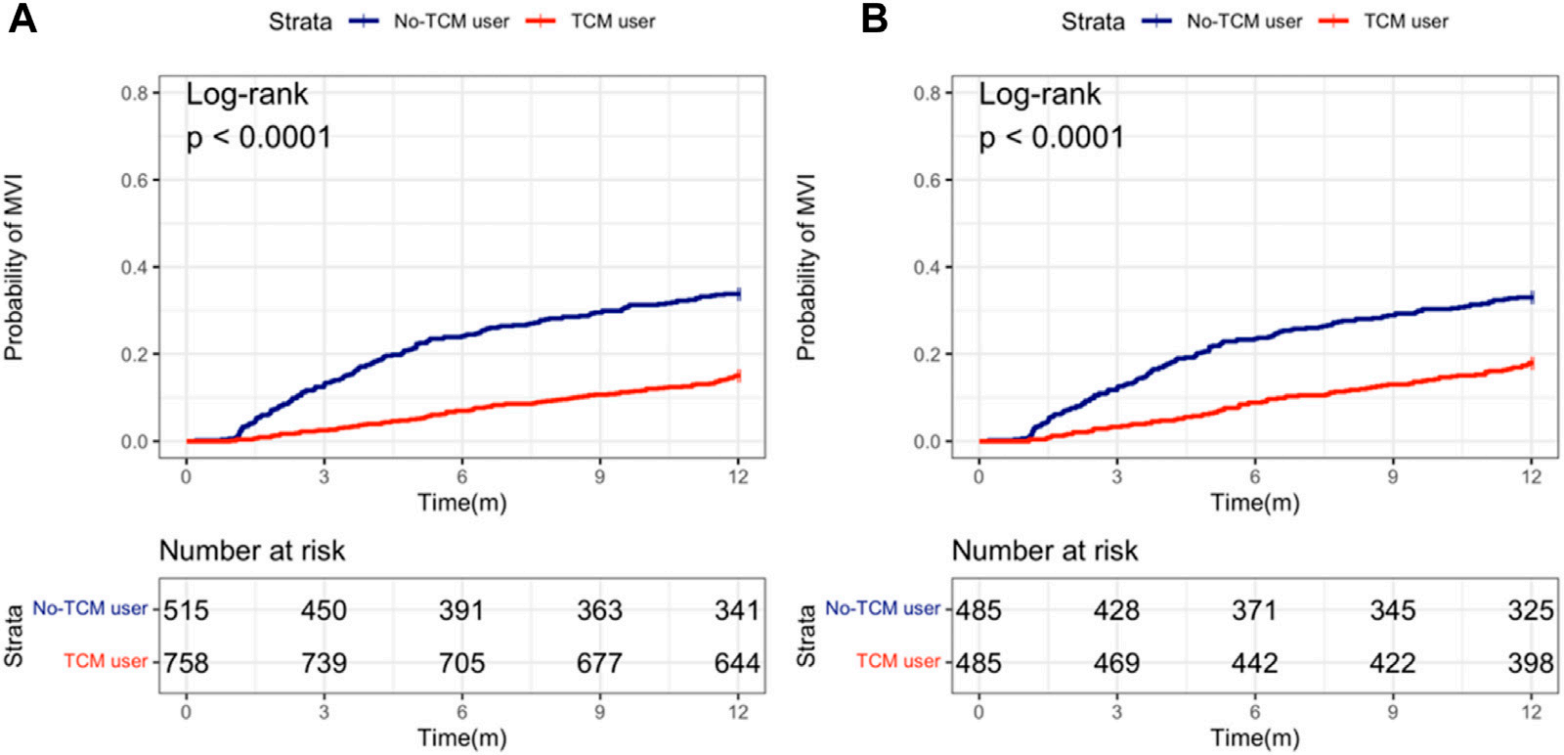
Probability of MVI occurring between TCM and non-TCM users in patients with hepatocellular carcinoma. **(A)** Before propensity score (PS) matching; **(B)** After PS matching.

Kaplan-Meier curve showed that before matching, the number of 1-year MVI occurrence in TCM-users group and non TCM-users group was 114 (15.0%) and 174 (33.8%) respectively, and after PS matching was 87 (17.9%) and 160 (32.9%), respectively. Regardless of PS matching or not, the 1-year MVI incidence in TCM-users was less than for non-users (Figures 2A,B).

**FIGURE 3:**
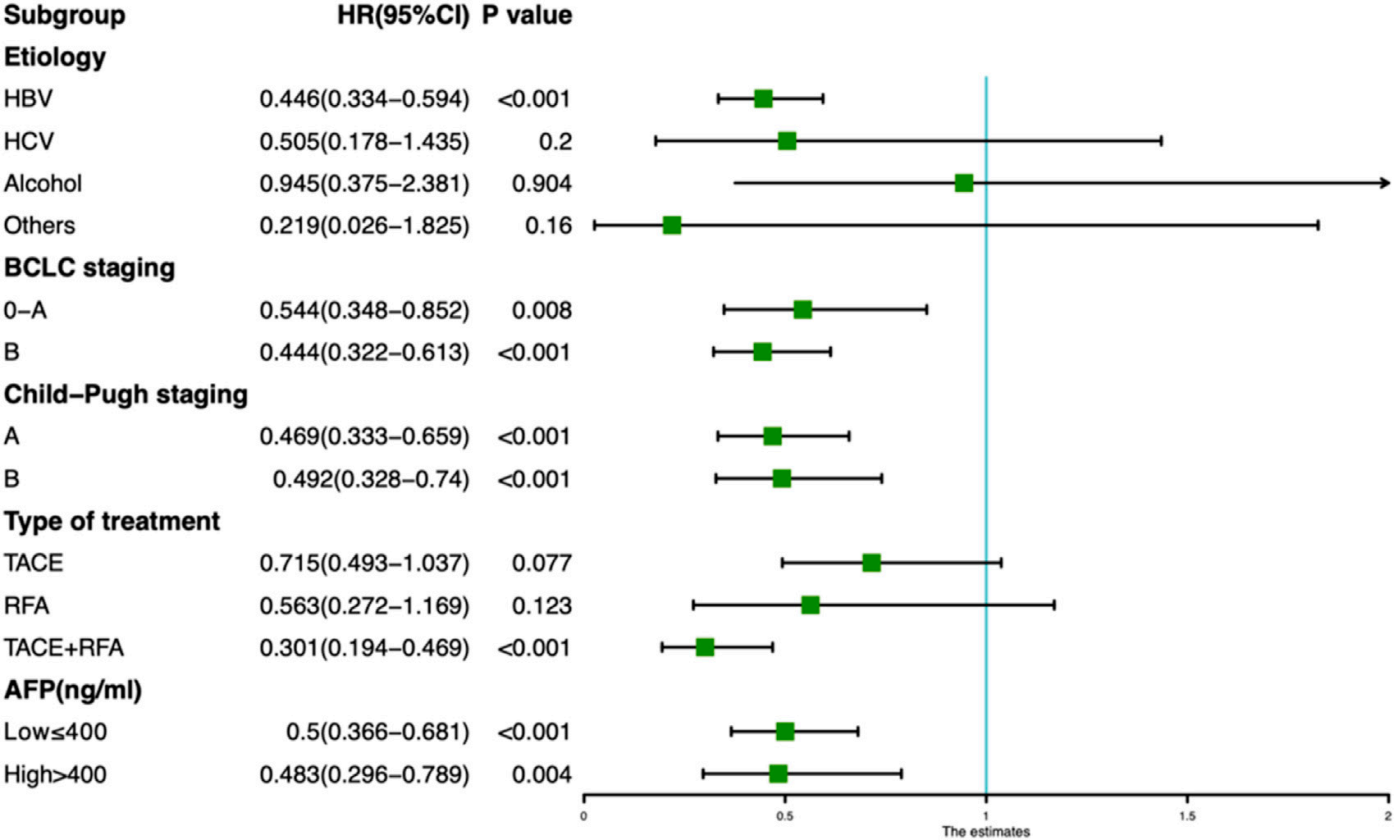
Forest plot showing the risk of developing MVI for TCM and non-TCM users in different subgroups of hepatocellular carcinoma patients.

In addition, we also analyzed the effects of taking TCM on the 1-year MVI incidence of HCC patients with different etiology, tumor stage, serum AFP level, liver functions and treatment type. The forest map revealed that the incidence of MVI in TCM users was less than in non-users, irrespective of the stage of HCC, good or poor level of liver function, high or low level of AFP. In addition, the incidence of MVI was significantly reduced for TCM users with HBV infection (HR = 0.446, 95% CI: 0.334–0.594) and TACE + RFA combination treatment (HR = 0.301, 95% CI: 0.194–0.469) (Figure 3).

### Comparison of mortality risks in individuals treated at various points in time and with various TCM modalities

**FIGURE 4:**
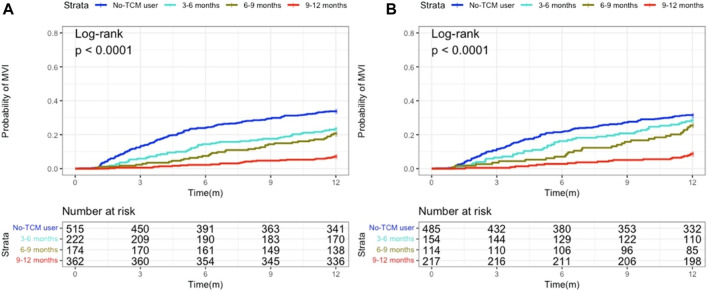
Kaplan-Meier analysis revealing the probability of MVI developing according to administration periods of TCM. **(A)** Before propensity score (PS) matching; **(B)** After PS matching.

Users of TCM were split into three categories based on how long they have been using Chinese medicine: 3 to 6, 6 to 9 and 9–12 months groups. Kaplan-Meier analysis demonstrated that the rate of occurrence of MVI was independent of PS matching and considerably lower in patients who were prescribed long-term TCM compared to non-users (Figure 4). The Cox model showed that, following PS matching, the probability of MVI emerging in the subgroup of patients taking TCM for 9–12 months was considerably lower than that in those not taking TCM. The Cox model also demonstrated that, as TCM use increased, the incidence of MVI decreased (Table 4).

**TABLE 4:** Risk of developing MVI according to the cumulative use (in months) of TCM among patients with hepatocellular carcinoma.

No. of TCM days	*n*	MVI events (*n* = 245)	Hazard ratio (95% CI) Crude^a^	*p*-value	Adjust^b^	*p*-value
Non-TCM users	485	153	1.00 (Reference)		1.00 (Reference)	
TCM users (TCM ≥3 months)						
3–6 months	154	44	0.851 (0.608–1.190)	0.345	0.869 (0.621–1.218)	0.416
6–9 months	114	29	0.720 (0.484–1.071)	0.105	0.742 (0.498–1.105)	0.142
9–12 months	217	19	0.232 (0.144–0.374)	<0.001	0.272 (0.168–0.440)	<0.001
*p*-value for trends				<0.001		<0.001

The results are presented as hazard ratios with corresponding 95% confidence intervals.

^a^ Crude HR, denotes the relative hazard ratio.

^b^ Adjusted HR, signifies the multivariate-adjusted hazard ratio. Specifically, the variables age, gender, tumor size, tumor numbers, alpha-fetoprotein (AFP), and type of treatment were adjusted for using the Cox model.

**FIGURE 5:**
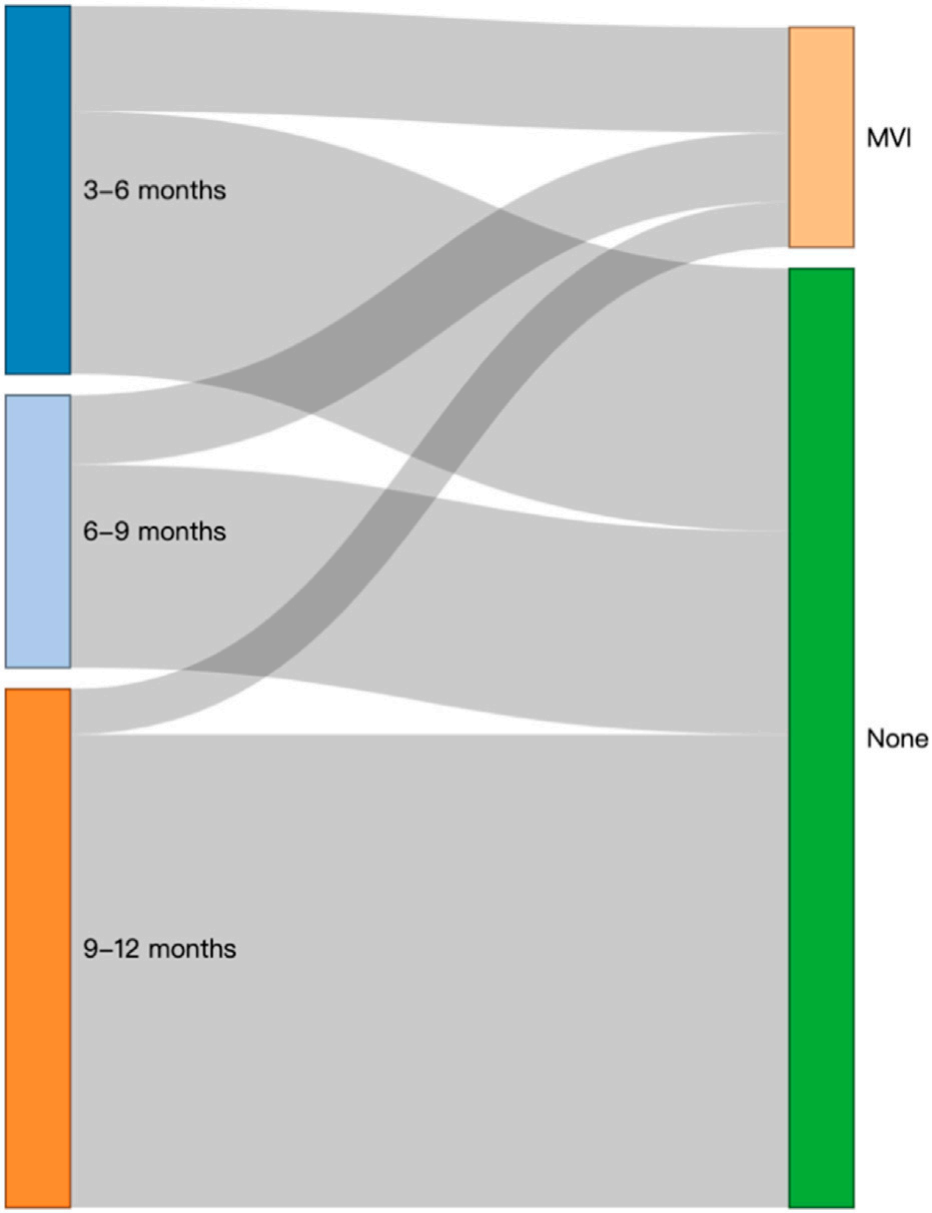
Sankey plot of the cumulative administration (months) of TCM.

In addition, the Sankey diagram intuitively showed the outcome and flow direction of MVI in patients who underwent different lengths of TCM treatment (Figure 5).

The analysis of different Chinese patent medicines taken in this study shows that Huier granule, Kanglixin capsule, Ganfule capsule, Jinlong capsule and Fufang Banmao capsule have significant protective effects on the rate of occurrence of MVI in HCC patients. The adjusted risk of MVI in 1 year is 0.402 (0.250–0.647), 0.402 (0.205–0.788), 0.541 (0.305–0.957), respectively 0.543 (0.325–0.907) and 0.594 (0.412–0.857) (Table 5).

**TABLE 5:** Risk of developing MVI according to the use of most common TCM among patients with hepatocellular carcinoma.

TCM prescription	*n*	MVI events (*n* = 245)	Hazard ratio (95% CI) Crude^a^	*p*-value	Adjust^b^	*p*-value
Non-TCM users	485	153	1.00 (Reference)		1.00 (Reference)	
TCM users (TCM ≥3 months)						
Huaier Granule	117	19	0.420 (0.261–0.676)	<0.001	0.402 (0.250–0.647)	<0.001
Kanglixin capsule	53	9	0.444 (0.227–0.869)	0.018	0.402 (0.205–0.788)	0.008
Ganfule capsule	74	13	0.447 (0.254–0.788)	0.005	0.541 (0.305–0.957)	0.035
Jinlong capsule	81	16	0.542 (0.324–0.906)	0.019	0.543 (0.325–0.907)	0.02
Fufang Banmao capsule	160	35	0.588 (0.408–0.848)	0.004	0.594 (0.412–0.857)	0.005
*p*-value for trends				<0.001		<0.001

The results are presented as hazard ratios with corresponding 95% confidence intervals.

^a^ Crude HR, denotes the relative hazard ratio.

^b^ Adjusted HR, signifies the multivariate-adjusted hazard ratio. Specifically, the variables age, gender, tumor size, tumor numbers, alpha-fetoprotein (AFP), and type of treatment were adjusted for using the Cox model.

## Discussion

So far, TCM has shown potential in slowing down tumor progression by downregulating VEGF-related signaling pathways. A number of studies have shown that the metabolite of TCM has significant therapeutic uses to suppress tumor angiogenesis in breast cancer, colon cancer, prostate cancer, glioblastoma and so on (Jiang et al., 2010; Yu et al., 2017; Zhu et al., 2017; Wang et al., 2018). The present study demonstrated that TCM can be used as a potential chemoprophylaxis to provide a lasting and significant inhibitory effect on tumor angiogenesis. Most studies focus on metabolites derived from TCM, rather than formulations, targeting tumor angiogenesis. It is worth noting that in the theory of TCM, the formula containing a variety of botanical drug combinations is the main form and are more commonly used as a treatment for cancer. Therefore, more research on the anti-tumor neovascularization of TCM should be conducted in order to explore further potential TCM therapeutic effects. Although one study has reported that a TCM metabolite can inhibit HCC angiogenesis by blocking the migration and invasion of vascular endothelial cells derived from tumor (Wei et al., 2017), there is a paucity of information on the potential effects of proprietary Chinese medicine at different tumor stages. This study analyzed the effect of proprietary Chinese medicine on MVI in patients diagnosed with HCC according to the clinical stage. The administration of proprietary Chinese medicine was shown to be a protective factor in reducing the rate of occurrence of 1-year MVI in patients with HCC, and any treatment of clinical tumor stages with TCM is related to the lower incidence of MVI.

In the early stage of tumor development, TCM can be used as a treatment to limit tumor progression. This study found that TCM significantly reduced the risk of developing MVI for patients treated with TACE combined with RFA. A randomized controlled trial reported that TCM combined with TACE could increase OS and PFS in patients with HCC, especially those with early HCC. TCM can effectively inhibit the proliferation and migration of HCC cells (Yang et al., 2021). In addition, a randomized, double-blind clinical trial showed that TCM can reduce the risk of recurrence of HCC after TACE and alleviate the postembolization syndrome (Xu et al., 2016). The curative effect of TCM is dependent on the treatment duration. Research has found that patients with HCC who have taken TCM for >36 months had a 90% lower risk of mortality than those who have not (Liu et al., 2019), findings in good agreement with our results, indicating that taking TCM for a long time will produce a stronger protective effect.

Recent research has shown that Fufang Banmao Capsule, a popular TCM, can be used as an adjuvant therapy for patients with HCC. It contains several active metabolites, including cantharidin, ginsenosides Rg1, Rg3, Rb1, Re, astragaloside A, and isofraxidin. Cantharidin is the main substance present in a concentration of at least 0.0175 mg/g (Naz et al., 2020). Studies have demonstrated that its active metabolite, astragaloside A effectively inhibits hepatocarcinogenesis by interfering with the NRF2/HO-1 signal pathway (Fang Gong et al., 2023). Another active metabolite of Fufang Banmao Capsule called Ginsenoside Rg3 has been found to inhibit tumor progression and metastasis in an orthotopic HCC transplantation mouse model by reducing VEGF levels and downregulating VEGF-R2 (Zhou et al., 2014). The results of studies on Fufang Banmao Capsule suggest that it is a preferred choice for adjuvant cancer therapy due to minimal adverse reactions and independent prediction of OS(Liu et al., 2020). Jinlong Capsule is a Chinese patent medicine derived from animal sources, commonly used in TCM for treating HCC. The capsule contains 17 amino acids and various bioactive peptides, including histidine, serine, arginine, glycine, aspartic acid, glutamate, threonine, alanine, proline, cystine, lysine, tyrosine, methionine, valine, isoleucine, leucine, and phenylalanin (Li C. et al., 2022). A meta-analysis conducted on the Chinese population showed that combining TACE with Jinlong Capsule significantly prolonged patients’ OS times and improved liver functions compared to TACE alone. This study also found that proprietary Chinese medicine had a protective effect on patients with compromised liver functions (Jia et al., 2020). Another meta-analysis evaluated the efficacy and safety of Jinlong Capsule as an adjuvant treatment for HCC, demonstrating its effectiveness in reducing adverse reactions such as leukopenia, gastrointestinal events, hepatotoxicity, and bone marrow suppression (Xu et al., 2020). Previous studies have shown that Kanglixin Capsule contains various metabolites, including costunolide, dehydrocostus lactone, aloe-emodin, rhein, emodin, physcion, bisdemethoxycurcumin, demethox-ycurcumin, and curcumin (Li and Kong, 2017). Curcumin is particularly noteworthy as it possesses strong anti-inflammatory and antioxidant properties (Lim et al., 2010). Research has indicated that when combined with metformin, curcumin can activate the mitochondrial pathway to induce apoptosis in cancer cells. Additionally, this combination treatment enhances the metastasis and invasion capabilities of HCC cells while promoting angiogenesis in HUVEC cells (Zhang H.-H. et al., 2018). UPLC-QTOF-MS analysis Ganfule Capsule showed that it contained 13 key metabolites. These metabolites include akebia saponin D, chlorogenic acid, amygdalin, 3′-deoxysappanone A, 10-O-Methylprotosappanin B, saikosaponin C and astragaloside I (Xu et al., 2022). Studies have shown that akebia saponin D inhibits the survival, proliferation, adhesion, migration and invasion of HCC cells in a concentration-dependent manner (Lu et al., 2019). The metabolites of Huaier Granule include proteoglycan, 2-Hydroxybutanoic acid, 2-Isopropyl-3-oxosuccinate, 2′-O-Methyladenosine, 3-Ethyl-1,2-benzenediol, and others. A multicenter study involving 1,044 patients demonstrated that Huaier Granule significantly improved postoperative intrahepatic metastasis (Chen et al., 2018). Additionally, Huaier Granule has shown promise as a prognostic tool for early-stage HCC patients treated with thermal ablation by increasing the PFS rate and reducing the rate of extrahepatic metastasis without notable side effects (Wang et al., 2021).

This study’s research methodology and data sources had several drawbacks including its retrospective nature. The clinical features of TCM and non-TCM users varied to some extent. There were still some biochemical discrepancies even after the patient groups were PS score matched to remove various confounding factors. Therefore, a prospective randomised controlled clinical trial should be conducted to validate the impact of TCM on MVI in HCC patients. The study excluded those individuals who were treated with decoction and only included patients who received proprietary Chinese medication. This study did not perform a thorough investigation because the majority of patients who consumed the decoction went to the clinic and only a tiny percentage of patients consumed it for longer than 3 months. Because there is no effective drug to treat HCC, there was no positive control drug. Most patients were in BCLC stage B and sorafenib should be considered as first-line treatment for advanced HCC patients. The patients in our study were not suitable for this treatment.

## Conclusion

In our retrospective cohort study, we found that adjuvant TCM treatment reduced the occurrence of MVI in patients with HCC. The use of TCM over an extended period showed a gradual decrease in the proportion of MVI, indicating its potential in preventing MVI in HCC patients. These findings suggest that TCM therapy is effective in preventing MVI in HCC patients. However, it is important to acknowledge the study limitations, including its retrospective design and reliance on observational data. To validate the efficacy and safety of TCM therapy in HCC treatment, future research should conduct prospective randomized controlled trials (RCTs) with robust experimental designs. Additionally, further investigations are needed to understand the mechanisms of action and identify optimal treatment protocols.

## Data Availability

The original contributions presented in the study are included in the article/Supplementary Materials, further inquiries can be directed to the corresponding author.

## Abbreviations

AFP, alpha-fetoprotein; ALB, albumin; ALT, alanine aminotransferase; AST, aspartate aminotransferase; BCLC, Barcelona Clinic Liver cancer; CRP, C-reactive protein; γ-GGT, γ-glutamyl transferase; HBeAg, hepatitis B e Antigen; HBV, hepatitis B virus; HCC, hepatocellular carcinoma; HGB, hemoglobin; HR, hazard ratio; INR, international normalized ratio; MELD, model for end-stage liver disease; PLT, platelet counts; PTA, prothrombin activity; WBC, white blood cell counts.
