# Supplementary material for: Effectiveness of adjuvant traditional Chinese medicine on macrovascular invasion in patients with hepatocellular carcinoma: a real-world propensity score-matched study
Source: Front Pharmacol. 2024 Feb 23;15:1353720. doi: 10.3389/fphar.2024.1353720 (PMC10923102; doi:10.3389/fphar.2024.1353720)
Supplement: Supplementary file 1 [file Table1.docx]

Table S1. Features of patients with HCC after 1:1 propensity score matching.

|  |  | Total  *n* = 970 (%) | Non-TCM users  *n* = 485 (%) | TCM users  *n* = 485 (%) | *p-*value |
| --- | --- | --- | --- | --- | --- |
| Patients background |  |  |  |  |  |
| Age, years  (mean ± SD) |  | 57.44 ± 10.07 | 57.14 ± 9.87 | 57.74 ± 10.27 | 0.349 |
| Gender | Female | 224 (23.1) | 110 (22.7) | 114 (23.5) | 0.819 |
|  | Male | 746 (76.9) | 375 (77.3) | 371 (76.5) |  |
| Family history of HCC | No | 937 (96.6) | 463 (95.5) | 474 (97.7) | 0.077 |
|  | Yes | 33 (3.4) | 22 (4.5) | 11 (2.3) |  |
| Smoking | No | 584 (60.2) | 300 (61.9) | 284 (58.6) | 0.325 |
|  | Yes | 386 (39.8) | 185 (38.1) | 201 (41.4) |  |
| Alcohol use | No | 607 (62.6) | 309 (63.7) | 298 (61.4) | 0.507 |
|  | Yes | 363 (37.4) | 176 (36.3) | 187 (38.6) |  |
| Hypertension | No | 703 (72.5) | 361 (74.4) | 342 (70.5) | 0.196 |
|  | Yes | 267 (27.5) | 124 (25.6) | 143 (29.5) |  |
| Diabetes | No | 753 (77.6) | 383 (79.0) | 370 (76.3) | 0.355 |
|  | Yes | 217 (22.4) | 102 (21.0) | 115 (23.7) |  |
| Hyperlipidemia | No | 893 (92.1) | 442 (91.1) | 451 (93.0) | 0.342 |
|  | Yes | 77 (7.9) | 43 (8.9) | 34 (7.0) |  |
| Coronary | No | 944 (97.3) | 469 (96.7) | 475 (97.9) | 0.320 |
|  | Yes | 26 (2.7) | 16 (3.3) | 10 (2.1) |  |
| Cirrhosis | No | 107 (11.0) | 61 (12.6) | 46 (9.5) | 0.151 |
|  | Yes | 863 (89.0) | 424 (87.4) | 439 (90.5) |  |
| Etiology | HBV | 750 (77.3) | 372 (76.7) | 378 (77.9) | 0.520 |
|  | HCV | 100 (10.3) | 55 (11.3) | 45 (9.3) |  |
|  | Alcohol abuse | 86 (8.9) | 39 (8.0) | 47 (9.7) |  |
|  | Other | 34 (3.5) | 19 (3.9) | 15 (3.1) |  |
| HBeAg | Negative | 495 (51.0) | 249 (51.3) | 246 (50.7) | 0.779 |
|  | Positive | 255 (26.3) | 123 (25.4) | 132 (27.2) |  |
|  | NA | 220 (22.7) | 113 (23.3) | 107 (22.1) |  |
| HBV-DNA, IU/mL | < 500 | 387 (39.9) | 178 (36.7) | 209 (43.1) | 0.113 |
|  | ≥ 500 | 363 (37.4) | 194 (40.0) | 169 (34.8) |  |
|  | NA | 220 (22.7) | 113 (23.3) | 107 (22.1) |  |
| MELD scores | | 4.14 (1.58, 6.68) | 4.39 (1.82, 6.77) | 3.93 (1.28, 6.59) | 0.167 |
| Child-Pugh Stage | A | 643 (66.3) | 322 (66.4) | 321 (66.2) | 1 |
|  | B | 327 (33.7) | 163 (33.6) | 164 (33.8) |  |
| BCLC staging | 0-A | 462 (47.6) | 220 (45.4) | 242 (49.9) | 0.177 |
|  | B | 508 (52.4) | 265 (54.6) | 243 (50.1) |  |
| Tumor multiplicity | Solitary | 564 (58.1) | 271 (55.9) | 293 (60.4) | 0.172 |
|  | Multiple | 406 (41.9) | 214 (44.1) | 192 (39.6) |  |
| Tumor size, cm | < 5 | 733 (75.6) | 354 (73.0) | 379 (78.1) | 0.073 |
|  | ≥ 5 | 237 (24.4) | 131 (27.0) | 106 (21.9) |  |
| Laboratory data |  |  |  |  |  |
| WBC (10^9^/L) |  | 4.46 (3.12, 5.94) | 4.59 (3.13, 6.00) | 4.23 (3.11, 5.80) | 0.089 |
| HGB (g/L) |  | 131.20 (115.05, 144.00) | 130.80 (115.00, 143.00) | 132.00 (115.20, 145.00) | 0.880 |
| PLT (10^9^/L) |  | 98.50 (62.40, 148.60) | 103.90 (64.60, 154.30) | 93.00 (62.00, 145.30) | 0.039 |
| ALT (U/L) |  | 31.00 (21.63, 50.38) | 32.50 (22.30, 53.60) | 30.30 (21.10, 47.40) | 0.065 |
| AST (U/L) |  | 36.40 (26.40, 57.05) | 38.60 (27.00, 62.60) | 35.10 (25.90, 53.30) | 0.011 |
| TBIL (µmol/L) |  | 16.70 (11.83, 23.28) | 16.60 (11.40, 23.30) | 16.80 (12.30, 23.10) | 0.847 |
| ALB (g/L) |  | 37.40 (33.12, 40.88) | 37.20 (32.70, 40.80) | 37.80 (33.50, 41.00) | 0.189 |
| γ-GGT (U/L) |  | 47.05 (29.72, 104.95) | 59.50 (33.60, 111.60) | 44.90 (26.80, 78.60) | < 0.001 |
| PTA (%) |  | 80.00 (70.00, 91.80) | 79.00 (69.30, 90.00) | 81.00 (70.40, 93.00) | 0.119 |
| INR |  | 1.09 (1.01, 1.19) | 1.10 (1.02, 1.21) | 1.09 (1.00, 1.18) | 0.022 |
| AFP (ng/mL) | < 400 | 753 (77.6) | 374 (77.1) | 379 (78.1) | 0.758 |
|  | ≥ 400 | 217 (22.4) | 111 (22.9) | 106 (21.9) |  |
| CRP (mg/L) |  | 3.20 (3.20, 12.97) | 3.20 (3.20, 17.70) | 3.20 (3.20, 6.10) | 0.001 |
| Type of treatment |  |  |  |  |  |
| TACE | No | 576 (59.4) | 283 (58.4) | 293 (60.4) | 0.556 |
|  | Yes | 394 (40.6) | 202 (41.6) | 192 (39.6) |  |
| RFA | No | 890 (91.8) | 444 (91.5) | 446 (92.0) | 0.907 |
|  | Yes | 80 (8.2) | 41 (8.5) | 39 (8.0) |  |
| TACE + RFA | No | 474 (48.9) | 243 (50.1) | 231 (47.6) | 0.48 |
|  | Yes | 496 (51.1) | 242 (49.9) | 254 (52.4) |  |
